# Implementing prehospital invasive arterial blood pressure monitoring in critically ill patients—a prospective observational first year analysis

**DOI:** 10.1186/s13049-025-01461-9

**Published:** 2025-09-02

**Authors:** Jakob Ule, Tobias Hüppe, Julian Thiel, Ulrich Berwanger, Thomas Schlechtriemen, David Conrad, Benedikt Merscher

**Affiliations:** 1 Department of Anesthesiology, Intensive Care and Pain Therapy, Saarland University Medical Center, Kirrberger Straße 100, Homburg (Saar), 66421 Germany; 2 Department of Anesthesiology, Surgical Intensive Care and Pain Medicine, Marienhaus Clinic St. Elisabeth Saarlouis, Kapuzinerstraße 4, Saarlouis, 66740 Germany; 3 Medical Director of Saarland Emergency Services and Fire Brigade Alerting (ZRF Saar), Saarpfalz-Park 9, Bexbach, 66450 Germany

**Keywords:** Arterial line, Invasive blood pressure monitoring, Prehospital, Critical care, Intra-arterial blood pressure, Post-resuscitation care, Emergency medicine

## Abstract

**Background:**

Exposure to hypotension is linked to increased morbidity and mortality. Invasive blood pressure (IBP) measurement might be superior to non-invasive blood pressure measurement in detecting hypotension. The feasibility of IBP in prehospital care for selected patients by specialized rescue teams has been demonstrated. Therefore, we tested the hypothesis that the implementation of prehospital IBP measurement is feasible in a German emergency system by emergency teams with limited exposure to critically ill patients.

**Methods:**

This single center study was conducted with two emergency physicians vehicles. Indications for IBP measurement were adults requiring airway management, catecholamine therapy or fluid resuscitation. IBP was performed using either direct or Seldinger technique. Physicians recorded the puncture attempts, cannulation sites, and techniques. Patients with IBP attempt were visited the first three days to report complications. Emergency physicians documented a reason if they decided not to perform IBP. Data were analyzed to find operational differences between IBP attempts and no IBP attempts and IBP success and failure. Multiple linear regression was used to measure the influence of prehospital IBP attempts on the on-scene time.

**Results:**

During the study period, 3887 emergency responses occurred, with 2.8% (n = 108) meeting IBP criteria. Reasons for an IBP were catecholamine therapy (74%), airway management (73%) and fluid resuscitation (51%). 68 (63%) of the patients meeting IBP criteria received an IBP attempt with a success rate of 88%. While difficult extrication (p = 0.002) and longer transportation time (p = 0.009) were associated with a high IBP attempt rate, IBP attempts in nursing homes were less often performed (p = 0.002). Most common reason for not performing IBP was a transport priority and poor puncturing condition. Multiple regression analysis showed IBP attempts prolonged the on-scene time by 7.4 min (p = 0.013).

**Conclusions:**

Prehospital IBP can be performed safely even by teams with limited exposure to critically ill patients, with low failure and complication rates across a wide range of indications. Based on these data, IBP measurement prolonged the on-scene time by 7.5 min. Even though exposure to critically ill patients is rare, teams should consider performing an IBP if indicated.

**Trial registration:**

Study was a part of the PHINIABP (PreHospital Invasive vs. Non-Invasive Blood Pressure) study and was registered with German Clinical Trials (ID DRKS00030477) and approved by the regional ethics committee (Ärztekammer Saarland, Saarbrücken, Germany, Identification Number 158/22, September 13, 2022). Written informed consent was obtained from patients or their legal representatives.

**Supplementary Information:**

The online version contains supplementary material available at 10.1186/s13049-025-01461-9.

## Introduction

Exposure to hypotension in critically ill patients is associated with increased morbidity and mortality [1]. Complications include acute kidney injury, myocardial damage and neurological impairment. The risk of such complications correlates with the duration and severity of hypotension [2–5].

Invasive blood pressure measurement (IBP) is superior to non-invasive blood pressure measurement (NIBP) in detecting hypotension [6, 7]. Inhospital IBP is one of the standard measurement procedures for critically ill patients and detects hypotension (mean arterial blood pressure (MAP) < 65 mmHg) more frequently compared to NIBP [8]. Moreover, during induction of anesthesia cumulative times with MAP < 65, 60, 50, and 40 mmHg are significantly shorter when IBP is used [9]. Consequently, patients with IBP receive more catecholamines compared to NIBP measurement [8].

However, in prehospital IBP measurement is rarely used although the number of critically ill patients in the prehospital emergency medicine is high. Nevertheless, prehospital IBP monitoring might be beneficial [10], if the cannulation does not delay or prevent other measures.

Prehospital feasibility studies show that the application of IBP measurement has a high success rate [11, 12] and does not delay the rapid transportation of patients [12]. However, the feasibility of IBP measurement has only been demonstrated in specialized teams in polytrauma [13] and resuscitation care [11] or in teams with many years of experience [12, 14].

Therefore, we tested the hypothesis that the implementation of prehospital IBP measurement in typical emergency systems in Germany is feasible. Specifically, this involves the general feasibility, the time required, the effectiveness and the complication rate of prehospital IBP monitoring.

## Method

### Study design

We performed a prospective, single-center observational cohort study at two physician-staffed emergency vehicles operated by Saarland University Medical Center (Homburg, Germany). Patients were enrolled during the initial 12-month implementation period of prehospital IBP. Study reporting was according to the STROBE cohort study statement (Supplement 1).

### Variables

The primary endpoint was the identification of factors associated with an attempted arterial puncture and reasons for not performing an IBP attempt. Secondary endpoints were comparison of successful vs. unsuccessful IBP attempts, complication rates of prehospital IBP attempts and analysis of prehospital time intervals of IBP attempts.

### Settings

The two emergency physician vehicles were staffed exclusively by anesthesiologists with an additional qualification in emergency medicine. The level of experience of the physicians deployed ranges from junior to senior physician with a minimum requirement of 2 years professional experience. In Germany emergency care is provided by trained emergency physicians who are called out in addition to paramedics in the event of life-threatening emergencies. The physicians are dispatched according to the indication catalog for emergency medical services 2023 by the German Medical Association [15]. The two vehicles cover an area with an average population density of 435 residents per square kilometers and a population of around 250,000 people, with a rate of 1.4 hospitals per 100,000 residents in the state.

Before the start of the study, the paramedics were introduced to the prehospital IBP measurement system in a one-hour training course. For the study period the pressure system was prepared at the beginning of the shift and remained usable for 24 h. A material bag for arterial puncture and IBP measurement was kept on the vehicle (puncture needles, disinfectant, compresses, plaster dressings, sterile gloves and fenestrated drapes). IBP module was integrated to the monitor system Corpuls3 (GS Elektromedizinische Geräte G. Stemple GmbH, Kaufering Germany). The material for the IBP measurement was identical to that used in the clinic (Table 1).

**Table 1:** Material bag for IBP measurement. IBP = Invasive Blood Pressure

Material for the Preclinical IBP Measurement
Corpuls C3 monitor with IBP module from GS Stemple corpuls^3^
Intermediate cable with GE Marquette socket (20 cm) from GS Stemple corpuls^3^
IBP adapter cable for Edwards transducer IBP-MQ-ED (400 cm) Edwards Lifesciences Services GmbH
Pressure system: TrueWave(3 cc)/200 cm, Edwards Lifesciences Services GmbH
Puncture cannulas: BD® Insyte-W™ 20 Ga 1.88 IN 1.1 × 48 mm
*Seldinger sets:* Arrow® Arterial Catheterization Set 20 Ga 8 cm Teleflex® Arrow® Arterial Catheterization Set 18 Ga 12 cm Teleflex®
Steril gloves GAMMEX® Latex, Ansell in Size 6; 6,5; 7; 7,5; 8; 8,5; 9
Fenestered shawl 75 × 90 cm, hole 9 cm, Raguse Gesellschaft für medizinische Produkte mbH
Suture material with needle Polyester 0 Mersilene™, Ethicon®
Needle holder: Sterile SUSI needle holder, AESULAP®
Local anesthesia Mecain® 10 mg/ml, PUREN Pharma GmbH & Co. KG
Cannula fixation plaster with foil view RUDAVEN®-universal, NOBAMED Paul Danz AG

IBP line was placed on scene or in the ambulance before transportation. Radial (direct needle puncture or Seldinger technique) or femoral artery (Seldinger technique) were permitted puncture locations with using ultrasound (Vscan Air, GE Healthcare) as a rescue option. A local anesthesia was administered to awake patients. For direct needle puncture spray disinfection was used, for Seldinger technique an additional fenestrated drape and sterile gloves. The catheter was secured with a fenestrated adhesive dressing. After connecting the arterial catheter, the pressure system was zeroed. The pressure transducer was attached to the upper arm at heart level. Stopping rules according to the procedure instruction for IBP attempts were more than two puncture attempts and a procedure duration of > 5 min.

All patients who underwent a successful or unsuccessful puncture were followed up for the first three days to monitor local infection or abnormal distal perfusion. Two out of four signs (pain, redness, increased temperature, and swelling) were sufficient to record a local infection. Only patients who were admitted to the study center were included.

### Participants

We defined indications for prehospital IBP; an attempt should be made if at least one individual criterion is met. Indications for IBP measurement were prehospital intubation, catecholamine administration (adrenaline, noradrenaline and dobutamine in any dose or cafedrine/theodrenaline ≥ 200/10 mg) or fluid administration ≥ 1.000 ml. Nevertheless, the final decision for or against an IBP was down to the emergency physician.

### Measurements

Data were used from prehospital records. Additionally, the emergency physicians recorded the number of puncture attempts, cannulation sites, surroundings and puncture technique on a study formula. Unsuccessful cannulations or cannulations not performed despite indication were noted in a free text format. Years of clinical practice as a physician were recorded. Response times were documented with (digital) time stamps by the emergency services dispatch center. Difficult extrication was defined as assistance by the fire department in a turntable ladder rescue, requesting a stretcher aid, vehicle extrication or opening doors. Medical history and long-term medication were taken from the patient files.

### Statistical Methods

Data were analyzed using IBM SPSS Statistics version 28.0.1.1. Missing data were handled using the pairwise deletion method. Normality was assessed with the Kolmogorov–Smirnov test. Group comparisons for mean values and standard deviations were performed using the student’s t-test for parametric data and the Mann–Whitney U test for non-parametric data. Categorical variables were analyzed using the chi-square test or Fisher’s exact test in case of n < 5. In the IBP group, differences in catheterization site and technique were assessed using the binomial test with an allocation probability of 0.5. Emergency on-scene time was analyzed with multiple linear regression to identify factors potentially influencing the duration. Confounders included difficult extrication, prehospital intubation, and prehospital resuscitation. A significance level of α < 0.05 was applied.

## Results

### Descriptive data

**Fig. 1:**
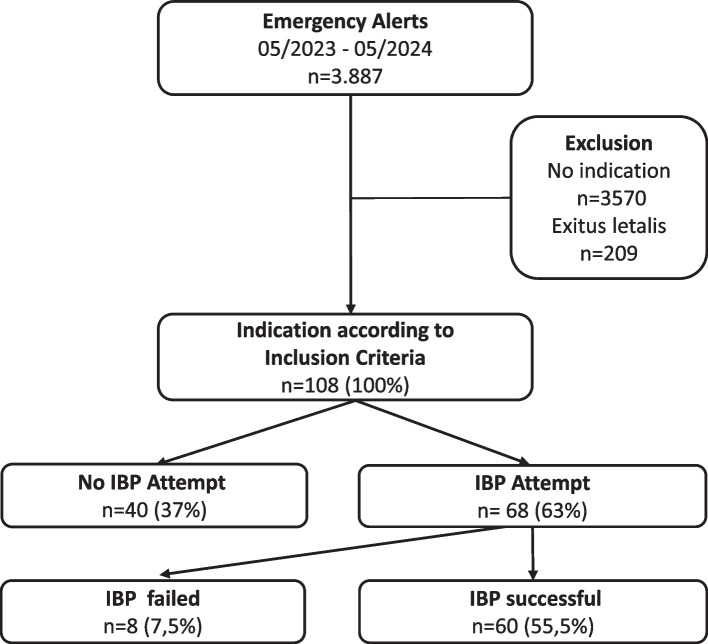
Flow diagram showing indications for IBP in relation to total number of emergency alerts

From May 2023 to May 2024, a total of 3,887 emergency responses were carried out. In 2.8% (n = 108) of all cases, at least one indication for IBP measurement was present (Fig. 1.). Criteria for IBP measurement were airway management (n = 79; 73%), catecholamine administration (n = 80; 74%) and fluid resuscitation (n = 55; 51%). A total of 54 patients (50%) had more than one indication. Overall, 68 patients (63%) received an IBP attempt with a success rate of 88% (n = 60). The most common clinical conditions associated with an indication for IBP measurement were cardiovascular failure and cardiopulmonary resuscitation, accounting for 44 cases (41%).

Demographic data, medical history and diagnosis categories are shown in Table 2. 9 out of 10 patients with a diagnosis of polytrauma had a leading traumatic brain injury (TBI) symptom. Septic/metabolic disease occurred more often in non-IBP group (*p* = 0.017) and sepsis occurred more frequently in nursing homes (*p* = 0.045).

**Table 2:** Demographic, medical history and diagnosis categories of patients with IBP attempt and no IBP attempt. Data are shown as n = (%) or mean ± SD. *P*-value for comparison categorial variables using the chi-square test or Fisher’s exact test (in case of n < 5) respectively with *p* < 0.05. *P*-Value for comparison of parametric data using an independent sample t-test with a *p* < 0.05. * = *p* < 0.05 statistically significant for comparison between groups. BMI = body mass index, CAD = Coronary Artery Disease, CKD = Chronic Kidney Disease, CNS = Central Nervous System, CPR = Cardio Pulmonary Resuscitation, PAD = Peripheral Artery Disease

	**Total**	**IBP-attempt**	**No IBP-attempt**	***P***-value
Total (%)	108 (100%)	68 (100%)	40 (100%)	
***Demography***				
Male(n)	65(60.2%)	41 (60.3%)	24(60%)	0.98
Female(n)	43 (39.8%)	27 (39.7%)	16(40%)	0.98
Age	66 ± 18	66 ± 19	67 ± 14	0.96
Height in cm	172 ± 9	173 ± 9	171 ± 9	0.39
Weight in kg	79 ± 17	78 ± 16	80 ± 19	0.68
BMI	26 ± 5	26 ± 5	27 ± 5	0.45
***Medical History***				
Hypertension	51 (47.2%)	34 (50.0%)	17 (42.5%)	0.19
Cerebrovascular	13 (12.0%)	5 (7.4%)	8 (20.0%)	0.08
CAD	21 (19.4%)	14 (20.6%)	7 (17.5%)	0.51
PAD	8 (7.4%)	6 (8.8%)	2 (5.0%)	0.75
CKD	13 (12.0%)	7 (10.3%)	6 (15.0%)	0.61
Coagulations in medication	20 (18.5%)	14 (20.6%)	6 (15.0%)	0.47
***Diagnosis category:***				
Cardiovascular	44 (41%)	32 (47%)	12 (30%)	0.11
*CPR*	*25 (57%)*	*18 (56%)*	*7 (58%)*	
*Cardiovascular failure*	*19 (43%)*	*14 (44%)*	*5 (42%)*	
Polytrauma	10 (9%)	8 (12%)	2 (5%)	0.32
CNS	15 (14%)	9 (13%)	6 (15%)	0.78
Pulmonary	10 (9%)	4 (6%)	6 (15%)	0.17
Abdomen	5 (4%)	4 (6%)	1 (2.5%)	0.65
Psychiatric	5 (4%)	4 (6%)	1 (2.5%)	0.65
Septic/metabolic	19 (18%)	7 (10%)	12 (30%)	0.017*

### Primary Endpoint

Operational data are shown in Table 3. The frequency of difficult extrication was significantly higher in the IBP attempts group (*p* = 0.002) and the location in the nursing home was significantly lower (*p* = 0.003). In 40 patients, no IBP attempt was made due to various circumstances. Transport priority was the most common reason (n = 11), followed by poor puncture conditions (n = 6), forgotten procedure (n = 5), priority on other measures (n = 3), poor team performance (n = 2), missing materials (n = 2), airway problems (n = 2), and deterioration during transport (n = 2). Additional reasons were lack of experience, significant patient improvement in post-CPR care, the palliative status of the patient, and the avoidance of additional equipment for an isolated patient (each n = 1). No feedback was given in four cases.

**Table 3:** Operational data of patients with IBP attempt and no IBP attempt. Data are shown as n = (%) or mean ± SD. *P*-value for comparison categorial variables using the chi-square test or Fisher’s exact test (in case of n < 5). Years of clinical practice was analyzed using Mann–Whitney-U-Test. *P*-Value for comparison of on scene time and prehospital transport time using an independent sample t-test. * = *p* < 0.05 statistically significant for comparison between groups. CPR = Cardio Pulmonary Pulmonal Resuscitation, CNS = Central Nervous System

	**Total**	**IBP-attempt**	**No IBP-attempt**	***P***-value
Total (%)	108 (100%)	68 (100%)	40 (100%)	
***Operational data:***				
Initial CPR	25 (23.1%)	18 (26.5%)	7 (17.5%)	0.29
Prehospital Intubation	79 (73.1%)	52 (76.5%)	27 (67.5%)	0.31
Nursing Home	15 (13.9%)	4 (5.9%)	11 (27.5%)	0.003*
Difficult extrication	13 (12%)	13 (19%)	0	0.002*
On scene time (min)	44 ± 16	48 ± 14	37 ± 16	0.001*
Prehospital transport time (min)	16 ± 12	19 ± 10	12 ± 13	0.009*
Years of clinical practice	8 ± 5	8 ± 5	7 ± 5	0.223

### Secondary Endpoints

The radial artery was cannulated in 82%, the femoral artery in 18% of all cases (*p* = < 0.001). The right side was more frequently used (69% vs 31%, *p* = 0.004). Overall, arterial catheters were placed more frequently in the ambulance (76.5%) than at the scene (23.5%, *p* = < 0.001). In twelve cases, the IBP placement took place at home, in two cases outdoors, and in one case in a doctor’s office. Number of successful and failed IBP attempts with catheterization location and site, puncture technique, place and number of attempts are shown in Table 4. In four cases, the unsuccessful IBP attempts were due to difficulties in advancing the wire or catheter. In two cases the puncture attempts were unsuccessful, in a further two cases the vein was punctured. Puncturing via Seldinger technique failed more frequently than direct puncturing (20% vs 6%, *p* = 0.26). Ultrasound-guided puncture was documented in only one case.

**Table 4:** Successful and failed IBP attempts with catheterization location and site, puncture technique, place and number of attempts. Data are shown as cases (%), number of puncture attempts as mean (range). P-value for comparison between groups using binomial test with an allocation probability of 0.5 for catheterization site, puncture technique and procedure. P-Value for comparison of puncture attempts using an independent sample t-test. * = *p* < 0.05 statistically significant for comparison between groups

	**IBP successful (100%)**	**IBP failed (100%)**	***p***-value
***Catheterization site:***			
Radial right	33 (55%)	6 (75%)	0.61
Radial left	16 (26.7%)	1 (12.5%)	
Femoral right	5 (8.3%)	0	
Femoral left	2 (3.3%)	1 (12.5%)	
Brachial right	2 (3.3%)	0	
Brachial left	2 (3.3%)	0	
***Puncture technique***			
Direct Punction	31 (51.7%)	2 (25%)	0.37
Seldinger technique	29 (48.3%)	6 (75%)	
***Procedure carried out***			
At home	10 (16.9%)	2 (25%)	0.4
In the rescue	47 (79.6%)	5 (62.5%)	
others	2 (3.4%)	1 (12.5%)	
***Number of puncture attempts***	1.4 (1–3)	1.9 (1–2)	0.02*

Among the patients admitted the study center no infections or perfusion deficits were detected during the first three days after admission.

The IBP attempt had significantly longer prehospital on-scene times (*p* < 0.001) as well as transport times (*p* = 0.009) compared to the non-IBP attempts.

The multiple linear regression analysis (n = 108) of the on-scene time revealed that IBP-attempt significantly affects the on-scene time by 7.4 min (*p* = 0.013) among all other factors shown in Table 5.

**Table 5:** Predictors for a delay in on-scene time. Multiple regression analysis with the cofounders Difficult extrication, prehospital intubation, prehospital cardiopulmonary resuscitation (CPR) and IBP-attempt were analyzed. * = *p* < 0.05 statistically significant for comparison between groups

Predictor	Regression Coefficient (min)	*p*-value
Difficult extrication	12.88	0.004*
Prehospital Intubation	6.43	0.047*
IBP-attempt	7.44	0.013*
Prehospital CPR	6.46	0.048*

## Discussion

This study’s results showed that prehospital IBP measurement in critically ill patients is feasible even for teams with limited exposure to critically ill patients in a wide range of emergency indications. The frequency of complications associated with the puncture appears to be negligible. Cardiovascular failure and the need for airway management with catecholamine administration were the most common reasons for arterial cannulation. Here in particular, pronounced hypotension is to be expected, which has fatal consequences and requires continuous monitoring of blood pressure and therapy [16].

Overall, the frequency of prehospital arterial puncture in our patients was very low: an indication for invasive blood pressure measurement was only given in every 35th emergency call. Strict IBP criteria confronted a broadly dispatched emergency physicians by the German indication catalog for emergency medical services 2023 [15]. This low-threshold dispatch model for emergency physicians is controversial and contributes to a lack of routine in managing critically ill patients. That could be a reason why arterial punctures were not performed in about one third of the emergencies in which IBP measurement was indicated.

Since the high non-IBP attempt rates were not attributable to years of work experience and we are investigating a new procedure, the rarity of the indication may have an influence on the attempt rates. IBP attempts were often forgotten due to their rarity. Since Butterfield et al. reported an increase in IBP rate over time as teams have become more familiar with the technique [13], lack of routine and high rates of decision against IBP might only be temporally. Motivation to perform the IBP may also have influenced the low IBP rates: two physicians accounted for 13 unattempted punctures, representing one-third of all missed puncture opportunities.

In nursing homes, we had a significantly lower rate of IBP attempts, but we saw no difference in age distribution between the groups. Beside that elderly trauma patients are under-triage [17], this result might be due to the judged futility.

The selected IBP procedure depended on the experience of the team members. The advantage of direct puncture is possibly the time advantage, since we do not use sterile gloves and covers. The Seldinger technique offers the advantage of easier puncture with the disadvantage of longer preparation, a sterile approach and requires adequate skills. It is widely accepted, that direct puncture is more demanding and associated with higher failure rate [18]. In opposite to the literature, our prehospital data showed a higher failure rate in the Seldinger technique. Because emergency physicians could freely select the puncture method, a selection bias is likely: in cases with poor vascular access and anticipated difficulties, they tended to choose the Seldinger technique, which may explain its higher failure rate. Nevertheless, the overall rate of failure rate was low and comparable to other studies [11, 12]. Our data showed that, despite this new procedure, the focus remained on life-saving measures and that teams with limited exposure to critically ill patients also discontinue the effort according to protocol. As outlined in the protocol, during the initial two months, the arterial pressure system was prepared every morning by the crew to reduce the procedure time. After considering the ecological aspects of not using the system 8 out of 10 times, we switched to the strategy of preparing the system on-scene. Assembling the IBP system gives team members with limited prior exposure a valuable opportunity to familiarize themselves with its components. In EMS systems that encounter critically ill patients more frequently, the preparation at the beginning of the shift with 24 h shelf life can reduce on scene time.

The IBP measurement results in an average time delay of about 7.5 min. The time delay is significant even when adjusting all tested confounders. It remains unclear whether the calculated delay is due to puncture attempts, or due to unmeasured confounders: our IBP times are not measured but calculated on the difference in total on-scene time (difference between documented arrival time at the patient’s site and departure time to the target clinic). Measured times suggest that the IBP alone are unlikely to prolong the overall prehospital interval with a mean preparation time of 3 min and cannulation time of 2 min [12]. Further studies must show whether the time difference is also detectable in larger patient collectives and if the delay has a negative impact on outcome. In the study, it was up to the medical team to decide whether IBP had a positive impact on the outcome. For this reason, arterial puncture was often not performed due to the priority of transport and short transportation times.

It must be considered whether this time advantage outweighs the potential risk of hypotension. Every provider should be aware that non-invasive blood pressure measurement has a low accuracy in hypotensive patients, especially in the prehospital settings [6, 7]. IBP measurement can be advantageous if correctly indicated, even if time plays a decisive role. Even a time delay of 7.4 min may not affect the outcome of patients. Of course, this must be considered on an individual basis, and more measures could be detrimental to trauma patients [19]. In our study polytrauma patients most frequently had leading TBI symptoms. In patients with severe TBI, prehospital hypotension has a negative effect on mortality, but time on scene does not [20] and the time to CT with prehospital IBP and intrahospital IBP were equivalent [21]. These findings are likewise reproducible in intubated patients presenting with suspected stroke [22]. It remains to be seen whether IBP measurement reduces the cumulative time with hypotension to hospital admission and whether it affects the outcome.

In our opinion, the advantages of prehospital IBP measurement in critically ill patients are the direct monitoring of fluid or catecholamine therapy, the visibility of the effects of malignant cardiac arrhythmia on blood pressure and aiding in decision-making. A synergistic combination with prehospital blood-gas analysis could unlock additional therapeutic options. We recommend preclinical arterial puncture in critically ill patients who require airway management or when severe hypotension with volume or catecholamine administration is expected. A therapeutic consequence should always be present and the focus of the measure, as a time delay must be expected. Arterial puncture during CPR should only be performed once all essential advanced life support interventions have been initiated. We emphasize that teams must be familiar with material and technique, because otherwise a high inhibition threshold will often prevent arterial punctures despite indication.

## Limitation

Our study has several limitations. We acknowledge that the exclusive inclusion of single center in this observational study may have led to a biased selection of results. The most important limitation is the lack of clinical outcome parameters after prehospital arterial cannulation. It is unclear whether IBP measurement in critically ill patients results in decreased morbidity or mortality. This question must be investigated in studies on larger patient groups. It is also unclear whether the time delay due the arterial cannulation was also due to selection bias, as the non-IBP group was recruited by deciding against an IBP intervention. Also, the severity of disease itself led to the study group allocation. A general higher medical expenditure can be assumed for these patients. The accessibility of the emergency side could also play a role and was not measured during the study period. Finally, it must be expected that prehospital arterial punctures are carried out under worse (hygienic) conditions than in the hospital. Whether prehospital puncture has actually led to such a low complication rate must be confirmed by studies on larger patient groups with longer follow-up.

## Conclusion

In conclusion, prehospital IBP measurement can be safely performed even by teams with limited exposure to critically ill patients, with low failure and complication rates across a wide range of indications. If the material and equipment are available and the indication is correct, the possibility of an IBP attempt should be considered even in standard EMS teams. The use of invasive blood pressure measurement must not prevent or delay life-saving measures.

## Supplementary Information

Supplemetary Material 1.

## Data Availability

The datasets analyzed during the current study are not publicly available but can be requested from the corresponding author upon reasonable request.

## Abbreviations

CNS: Central nervous system
CPR: Cardio pulmonal resuscitation
IBP: Invasive blood pressure
MAP: Mean arterial pressure
NIBP: Non-invasive blood pressure
TBI: Traumatic brain injury
